# Botanical Origin and Biological Properties of Honey and Propolis from Cuautitlan, State of Mexico, Mexico

**DOI:** 10.3390/antiox13070874

**Published:** 2024-07-20

**Authors:** Jose Juan Alcivar-Saldaña, Marco Aurelio Rodriguez-Monroy, Liborio Carrillo-Miranda, Maria Margarita Canales-Martinez

**Affiliations:** 1 Laboratorio de Farmacognosia, Unidad de Biotecnología y Prototipos (UBIPRO), Facultad de Estudios Superiores Iztacala, Universidad Nacional Autónoma de México (UNAM), Avenida de los Barrios Numero 1, Colonia Los Reyes Iztacla, Tlalnepantla de Baz CP 54090, Edo. Mex., Mexico; bio.alcivar@gmail.com; 2 Laboratorio de Investigación Biomédica en Productos Naturales, Carrera de Medicina, Facultad de Estudios Superiores Iztacala, Universidad Nacional Autónoma de México (UNAM), Avenida de los Barrios Numero 1, Colonia Los Reyes Iztacla, Tlalnepantla de Baz CP 54090, Edo. Mex., Mexico; dr.marcorodriguezmonroy@gmail.com; 3 Módulo de Apicultura, Centro de Enseñanza Agropecuaria (CEA), Facultad de Estudios Superiores Cuahutitlan, Universidad Nacional Autónoma de México (UNAM), Carretera Cuautitlán-Teoloyucan km. 2.5, Col. San Sebastián Xhala, Cuautitlán Izcalli CP 54714, Edo. Mex., Mexico; liboriocami@gmail.com

**Keywords:** propolis, honey, pollen, phenols, flavonoids, antioxidant capacity

## Abstract

Beekeeping is an activity that generates various products, mainly honey and propolis, with different biological activities that are studied extensively using various methodologies. The influence of various phenolic compounds, such as phenols and flavonoids, which are synthesized and concentrated differently in each product depending on the melliferous flora and sources of resources, on the manufacture of propolis or honey has been investigated. However, the analysis of these products has been performed separately and is outdated in time, and depending on the area and the flowering periods, different crops may be harvested. The analysis of the honey and propolis produced in Cuautitlan, State of Mexico, in the high plateau beekeeping zone, for a period of four years, both in the dry and rainy seasons, was proposed to determine the botanical origin of the honey and propolis. The primary pollen type in both honey and propolis was from *Brassica rapa*. Physicochemical tests were conducted, revealing higher concentrations of antimicrobial activity in the dry season than in the rainy season. Honey, propolis, and a vegetation extract showed activity against *S. aureus*, while only honey had an effect on *E. coli* in both seasons. For antifungal activity, only propolis collected in the rainy season had this activity. The biological properties of these products are closely related to the flora that varies both annually and between seasons, influencing the concentrations of phenolic compounds, as well as the biological activity of honey and propolis.

## 1. Introduction

Beekeeping has been an activity that has accompanied humanity throughout its history, which has benefited from its products such as honey and propolis, resources that are consumed mainly for their nutritional and medicinal properties. Currently, the use of honey and propolis has been resumed with a more methodical approach and directed to verify and certify the nutritional and biomedical properties of these products, with the conducting of research that has characterized the bactericidal, antiviral, and antifungal potential of these products, which, when corroborated, has been able to promote honey and propolis as an effective alternative against various species of pathogenic bacteria with multiresistance to antibiotics [1,2].

The physical and chemical composition of honey and propolis is closely related to the biotic and abiotic factors in which bees work, such as the blooms of different species throughout the year and the climatic conditions present in the area, which, together with the health and strength of the colony, allow the incorporation of enzymatic compounds that result in the production of products of different compositions and qualities [3]. In general, honey is composed of 17% water, 38.4% fructose, 30.3% glucose, 1.3% sucrose, 8.7% other disaccharides, 0.57% gluconic acid, 0.43% organic acids, 0.14% lactones, 0.17% minerals, and 0.04% nitrogen [4,5]. This composition, together with the fact that honey is a saturated solution and a highly osmotic medium, does not allow bacterial growth, which, together with the different organic acids, which are phytochemical components, allows this product to have diverse activities, such as antifungal, antibacterial, and antiviral properties [4,5,6].

With regard to the chemical composition of propolis, it is known that it is composed of 20–30% waxes, 40–50% resins and aromatic balsams, 5–10% essential oils, 4–5% pollen, and 10–30% mechanical impurities, which make up a mixture to which the bees not only add wax but also saliva with different enzyme complexes, such as 3-glycosidase, which generates the hydrolysis of glycoside flavonoids into flavonoid aglycones; this promotes a high variation in chemical composition, which is why up to 300 different compounds have been characterized, such as polyphenols, phenolic acids, esters, aldehydes, alcohols, ketones, terpenoids, amino acids, and inorganic compounds, which allows propolis to be designated as an excellent antioxidant with antibacterial, antifungal, and antiviral properties [7,8,9,10,11,12,13].

Therefore, several studies have characterized the chemical composition of these products by means of HPLC analysis, highlighting that both products have a remarkable phenolic profile. As mentioned above, the chemical composition and physical properties are closely related to the melliferous flora, so botanical characterization becomes relevant since, with this information, we can assume the possible contributions of phytochemicals and classify the products by their main botanical contribution [3,14].

Various methods have been implemented in the identification of the botanical origin of honey and propolis, ranging from the observation of the flora visited by the honeybee to more specialized techniques, such as palynological analysis, which allows recognition with greater precision than previous methods [3]. With the establishment of the botanical origin, which sources and inputs are used by honeybees for the elaboration of these products is established, information that in turn allows the classification and assessment of their economic potential, as well as their possible biological properties [3,15,16,17].

In terms of honey production, Mexico has great floral and climatic diversity, which promotes the production of honey of various compositions, colors, smells, and flavors with high nutritional, economic, and medicinal value [3]. Therefore, these differences have led to the regionalization of the country into five defined beekeeping regions based on the botanical origin of the honey produced in each region [3,18,19]. However, national per capita consumption is low, and honey production is mainly destined for exports, ranking Mexico among the top ten producers and exporters at the international level [20,21].

However, most of the honey and propolis produced in the country are characterized by noncompliance with export parameters due to noncompliance with good beekeeping practices, as well as laws and regulations in force, which, together with the parameters requested in international regulations, decrease the quality and economic value of Mexican production [21]. Thus, rejected honey is collected and mixed to form what is called the Mexican blend and exported at a lower cost, affecting the added value assigned to this product [3,21].

The scarcity of Mexican propolis, similar to honey, is due to various factors, such as low technology and a lack of knowledge of the various properties of this product, which has been shown in various investigations not only to be of good quality but also, depending on the beekeeping region, to have different compositions, colors, and properties, highlighting its antibacterial and antifungal actions [18]. However, for both propolis and honey, no studies have related their biomedical properties and phytochemical characteristics to their botanical origin [18,22].

Therefore, this research aimed to determine both the botanical origin and physicochemical characteristics of honey and propolis from Cuautitlan, State of Mexico, Highlands Region, Mexico. It is hoped that these characteristics are related to the biological activity of these products and that the knowledge generated will promote an added value that will allow the development of the apicultural activity, reflected in greater and better honey and propolis production that will boost exports and national per capita consumption.

## 2. Materials and Methods

The honey and propolis samples were provided by the Department of Apiculture of the Facultad de Estudios Superiores, Cuautitlan campus 4 (FES-C), located in the municipality of Cuautitlan, State of Mexico, Mexico, in the Altiplano apicultural region at coordinates 19°41′23.2″ N 99°11′51.9″ W for a period of four years from 2019 to 2022.

The samples were donated by FES-C, which provided 1 kg of honey that was packaged in hermetically sealed glass jars and stored at room temperature. Honey collection was performed in April, known as the dry season, and a second collection occurred in November, known as the rainy season, with sampling from 2019 to 2022 [23].

Propolis samples were collected between November and February 2019 and 2022 using Multihive^®^ brand propolis collectors (PCs) installed in the hives. The collectors, together with the samples obtained, were frozen at −4 °C because volatile compounds such as terpenes are preserved at this temperature, and in turn, propolis behaves fragile and brittle, being easily extracted from the PC by cryofracture and obtaining what is known as raw propolis (RP) [17]. The RP was manually cleaned of impurities such as pieces of wood, insect remains, or other artifacts foreign to the propolis composition at room temperature; once cleaned, the resulting material was placed in glass containers and frozen at −4 °C for preservation [17]. In turn, honey and propolis samples collected in April and November 2019 to 2022 were numbered from 1 to 8 with the prefix M for honey and P for propolis.

**Figure 1:**
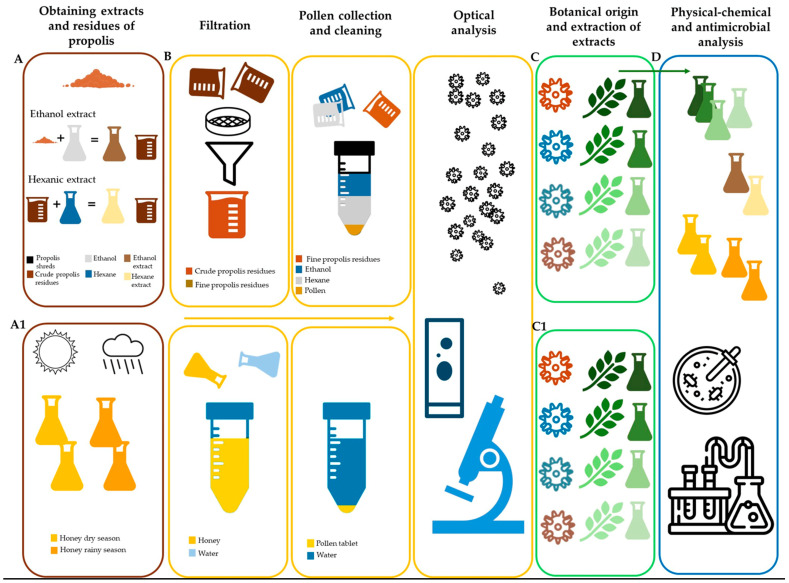
Flow chart of the various methods implemented in the analysis of honey and propolis samples. (**A**) Maceration of propolis, (**A1**) Honey samples, (**B**) extraction of pollen from propolis and honey (pollen analysis), (**C**) determination of the main vegetation species in propolis and maceration to obtain extracts, (**C1**) determination of the main vegetation species in honey and maceration to obtain extracts, (**D**) chemical and biological analysis of honey, propolis and vegetation of the main pollen types.

For the analysis of both honey and propolis samples, different methodologies were used so that from the same sample, whether propolis or honey, resources such as ethanolic and hexanic extracts useful for different chemical and biological analysis methodologies could be obtained. On the other hand, the residues resulting from the maceration processes were subjected to various treatments to extract the pollen grains for palynological analysis and to determine the melliferous and propoliferous flora (Figure 1).

### 2.1. Analysis of Honey and Propolis

Honey samples were analyzed for color, moisture, pH, Brix, and fructose content. Both moisture and Brix were obtained using an Atago model RePo-4 refractopolarimeter. Color was measured with a honey color photometer (Hanna model HI 96785), and pH was measured with a potentiometer (Hanna model HI2030-01). For propolis, physical characteristics, such as color, aroma, and consistency, were determined as specified in official standards [24].

### 2.2. Propolis Processing

The propolis samples were processed using the maceration method until exhaustive extraction of the phytochemical components that constitute them was reached, considering the polarity of each constituent, in such a way that the solvent for the extraction of phenols and flavonoids was 70% methanol in a 1:5 ratio for 72 h. Three maceration phases and three filtration phases were required to obtain a methanolic extract of propolis, which was dried using a rotary evaporator at 40 °C, and soft propolis (SP) was obtained [25]. In turn, the residues obtained from the previous process were reincorporated into a maceration process similar to the previous process using hexane as the solvent, the sample was degassed, and the waxes were obtained in a hexane extract. Both extracts were stored frozen at −4 °C for preservation [25,26].

### 2.3. Obtaining Pollen for Palynological Analysis of Honey and Propolis

The residues obtained from the extraction process described above were resuspended in 50 mL of 96% ethanol. Once the solution was homogenized, it was filtered through sterile gauze many times to retain the greatest amount of residue remaining in the sample, and the solution obtained was placed in 50 mL centrifuge tubes and gavaged with ethanol, if necessary [15,27]. The samples were centrifuged at 2500 rpm for 10 min, after which the supernatant was discarded, and the pellet was retained, which appeared as a conglomerate of resins, waxes, and pollen [15,27].

The obtained pellet was resuspended in a double phase of ethanol–hexane solvent to remove the pollen from the resins and waxes, and once homogenized, the sample was again centrifuged at 2500 rpm for ten minutes [15,27]. Afterward, both the resins and waxes were dissolved and retained in the double phase of the solvent, allowing the formation of a pellet composed mostly of pollen [15,27,28].

The extraction of pollen from honey was performed by dissolving 10 g of honey in 50 mL of distilled water and centrifuging the sample at 2500 rpm for 10 min, after which a tablet containing the pollen was obtained [28].

Both propolis and honey pellets were resuspended in 100 µL of glycerogelatin with basic fuchsin to determine not only the pollen content but also the quality of the propolis and honey. Once homogenized, the pollen–glycerogelatin samples were mounted on slides, forming semipermanent preparations that were analyzed under an optical microscope to determine the botanical origin of each of the samples [28,29].

### 2.4. Palynological Analysis

The palynological analysis of both propolis and honey samples was performed using the methodology proposed by Trigo et al. [29] by determining the different pollen structures and identifying the families, genera, and species to which they belong. Once the botanical origin of the pollen grains contained in both products was determined, the pollen percentage was established by counting 500 pollen grains in four equidistant transects marked on the preparation, differentiating the families, genera, and species previously determined [15,28]. With this information, the honey samples were classified as monofloral or multifloral using the classification proposed by Louveaux et al. [15], which establishes that more than 45% of the pollen is predominant pollen, 16–45% is secondary pollen, 3–15.9% is important minority pollen, and less than 3% is minority pollen.

With the determination of the botanical origin of honey and propolis based on the main pollen types, samples were collected in the field, dried, and macerated for exhaustive extraction in methanol for 72 h to obtain a methanolic extract to assess the physical and chemical activities and possible antibacterial and antifungal activities [25].

### 2.5. Physicochemical Analysis

Both honey and propolis were evaluated for physicochemical parameters such as total phenol content using the method reported by Singelton et al. [30], with modifications; total flavonoid content using the Down method reported by Ramamoorthy et al. [31]; antioxidant capacity using the method described by Okusa et al. [32]; organic acid quantification using the method described by González and Peñalosa [33]; ascorbic acid quantification using the Jagota and Dani method [34]; the quantification of reducing carbohydrates using the Nelson–Somogyi technique reported by González and Peñalosa [33]; and the permanganometric determination of hydrogen peroxide using the method described by Chang [35].

### 2.6. Evaluation of Antimicrobial and Antifungal Activities

The antimicrobial and antifungal activities of honey samples, propolis, and plant extracts belonging to the species producing the main pollen types were evaluated through palynological analysis. The qualitative evaluation of both bacteria and yeast fungi was performed through the Kirby–Baüer agar diffusion method using Sensi-Discs impregnated with 4 mg of propolis, honey, or the extract for analysis, as well as positive control Sensi-Discs impregnated with 25 µg of nystatin for yeast fungi and 25 µg of chloramphenicol for bacteria. The bacterial species used for antifungal activity assessments were *Staphylococcus aureus* ATCC 25923 Gram (+), *Escherichia coli* clinical case Gram (−) and the yeast fungus *Candida albicans* ATCC 10231, all of which are available in the ceparium of the Pharmacognosy Laboratory of the Biotechnology and Prototypes Unit of the Facultad de Estudios Superiores Iztacala, [FES-I] UNAM [36].

### 2.7. Statistical Analysis

With the results obtained, Student’s *t* test was applied to each of the analyses performed to determine whether there was a significant difference between seasons.

## 3. Results

### 3.1. Physicochemical Analysis

#### 3.1.1. Honey

**Figure 2:**
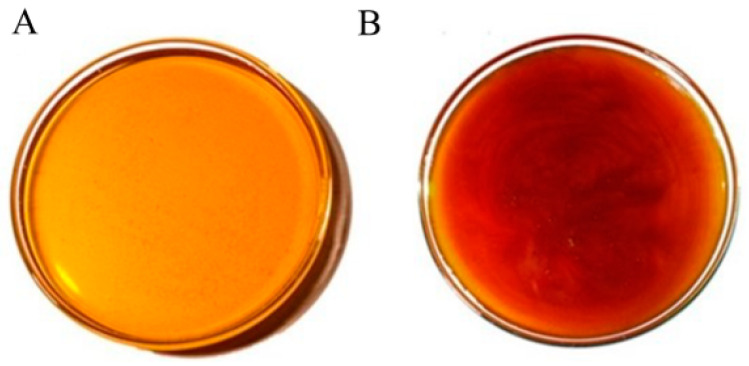
Changes in coloration between the honey collected at FES-C. (**A**) Collection in the dry season; (**B**) collection in the rainy season.

The honey obtained in Cuautitlan presented two colors according to the Pfund scale, with an average of 83.25 mm, resulting in light amber for the sample collected in the dry season, while for the honey collected in the rainy season, an average of 106.75 mm was obtained and recognized as amber colored (Figure 2). This indicated a significant difference between seasons (*p* < 0. 01), as the rainy season honey was darker than the dry season honey, with an average moisture content of 19.3%, with no significant difference (*p* > 0.05); for pH, the average was 3.6, with no significant difference between seasons (*p* > 0.05); in terms of Brix, the average was 82.3%, with no significant difference between seasons; in terms of fructose content, the average was 54.64%, with no significant difference (*p* > 0.05) (Table 1).

#### 3.1.2. Physical Characteristics of Propolis

**Figure 3:**
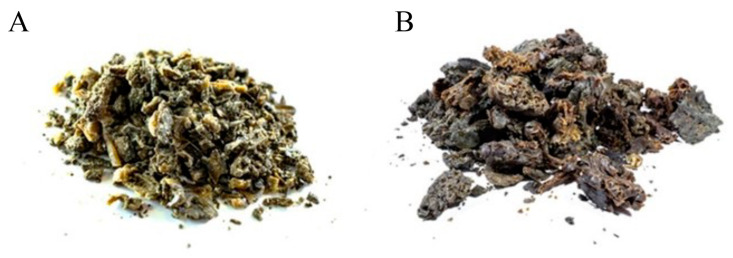
Physical characteristics of propolis collected during both the rainy and dry seasons. (**A**) Propolis collected in the dry season; (**B**) propolis collected in the rainy season.

The propolis samples obtained in both the dry and rainy seasons were small in shape with less than 10% impurities due to the use of PC and were green in color, while the propolis collected in the rainy season had brown inclusions, as shown in Figure 3. The propolis from both seasons was rubbery and sticky. The aroma of propolis is usually different because propolis samples collected in the rainy season usually have a stronger aroma.

### 3.2. Propolis Processing

#### 3.2.1. Obtaining the Propolis Extract

The use of maceration to perform an exhaustive extraction of propolis components made it possible to obtain extracts useful for physicochemical analysis, such as a methanolic extract and a hexanolic extract. The average yield of the ethanolic extract was 22.33% for the dry season, with P4 having the highest yield (25.21%), while for the rainy season, the average yield was 32.86%, with P5 having the highest yield (49.27%). There was no significant difference between seasons for this extract (*p* > 0.05) (Table 2). For the hexane extract (wax content), an average yield of 10.44% was obtained in the dry season, with P8 having the lowest percentage of 9.67%, and in the rainy season, the average percentage was 7.24%, with P3 having the lowest percentage of 6.19% (Table 2). There was a significant difference between seasons (*p* < 0.02), with the rainy season having the lowest concentration of waxes. For conservation and subsequent analysis, both extracts were stored at −4 °C.

#### 3.2.2. Pollen Collection for Palynological Analysis

**Figure 4:**
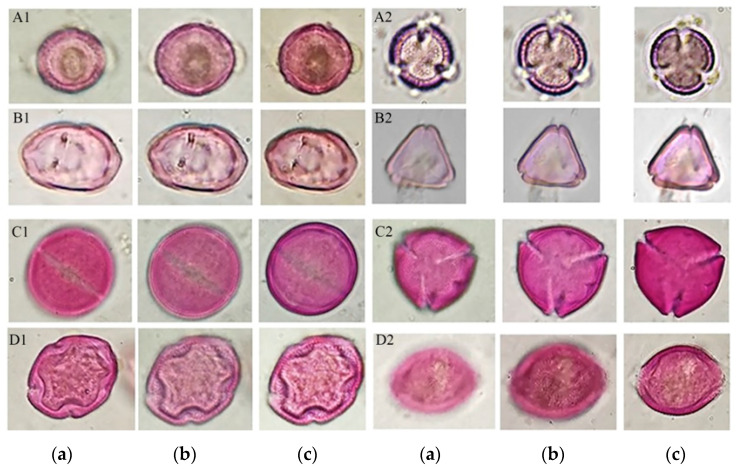
Optical sections of some pollen types are shown. Main pollen type *Brassica* in equatorial view (**A1 a–c**); main pollen type *Brassica* in polar view (**A2 a–c**); secondary pollen type Myrtaceae in equatorial view (**B1 a–c**); secondary pollen type Myrtaceae in polar view (**B2 a–c**); and minority pollens with allergenic potential: *Ricinus communis* in equatorial view (**C1 a–c**); *Ricinus communis* in polar view (**C2 a–c**); *Fraxinus uhdei* in equatorial view (**D1 a–c**); and *Fraxinus uhdei* in polar (**D2 a–c)**.

In turn, this process disintegrated the conglomerate of resins and waxes, allowing the release of the pollen content, among other artifacts present in the sample, so that the residues obtained were incorporated into a methodology of pollen extraction for palynological analysis, which allowed for obtaining pollen in suitable conditions for palynological analysis, observing different morphological structures useful in the identification of pollen types, as shown in Figure 4.

### 3.3. Palynological Analysis

#### 3.3.1. Melisopalynological Analysis

The palynological analysis of the honey samples revealed that *Brassica rapa* L. (IZ-TA-3812) pollen was the predominant pollen type, with an average of 50.34% for samples collected in the dry season, while those collected in the rainy season represented 41.13%, showing a significant difference (*p* < 0.01), with the rainy season having the greatest number of species. In turn, as a secondary pollen type, the species *Melaleuca citrina* (Curtis) Dum. Cours (IZTA-3820) represented 12.44% of the pollen collected in the dry season and 21.56% of the pollen collected in the rainy season. For the main minority pollen type, we considered *Ricinus cummunis* L. (FESC-6294) for the dry season, with 3.52%, and *Fraxinus uhdei* (Wenz.) Lingelsh (IZTA-42812) had a value of 1.23% for the rainy season, as shown in Table 3.

With the determination of the pollen types and their respective percentages, the honey samples could be classified as monofloral or multifloral; in the dry season, three monofloral samples (M1, M5, and M7) and one multifloral sample (M3) were obtained, and in the rainy season, all the samples were multifloral [15].

#### 3.3.2. Palynological Analysis of Propolis

The palynological analysis of the propolis samples revealed that the predominant pollen was the pollen of *B. rapa*, with an average of 57.31% for the dry season and 41.13% for the rainy season, with no significant difference between seasons (*p* > 0.05). The main secondary pollen was considered the pollen of the species *M. citrina* for both seasons, with averages of 12.44% and 21.56%, respectively. The main secondary pollen type was *R. communis* for propolis collected in the dry season, with 4.41%, and *F. uhdei* for propolis collected in the rainy season, with 4.22%, as shown in Table 4.

### 3.4. Flowering Calendar

With the determination of pollen percentages, the most abundant source plants of pollen were collected, three specimens were herborized for the determination of the species by the IZTA Herbarium, and others were collected for drying and obtaining methanolic extracts. The work of species determination resulted in 36 species corresponding to 16 families with which a flowering calendar was designed, which defines the available resources in both the dry and rainy seasons. A greater number of resources was observed in the rainy season than in the dry season, highlighting the Asteraceae family (Table 5) [37,38].

At the same time, by obtaining the pollen percentages, four species were defined for the analysis of their properties, two of which were considered the main pollen types and two of which were species with allergenic potential, namely, the defined species *B. rapa* (V1), *M. citrina* (V2), *R. communis* (V3), and *F. uhdei* (V4). Methanolic extracts of each of the plant species were obtained from completely dried plant material obtained in the field using the maceration method with methanol as a solvent for physicochemical and antibacterial analyses [25].

### 3.5. Physicochemical Analysis

#### 3.5.1. Total Phenols

The mean concentration of total phenols in honey was 1.84 ± 0.24 mg AGe·g^−1^ (FW) for the dry season and 1.89 ± 0.30 mg AGe·g^−1^ (FW) for the rainy season, with no significant differences between the two seasons (*p* > 0.05). In the case of propolis, the concentration was 53.28 ± 15.93 mg AGe·g^−1^ for the dry season and 73.56 ± 42.28 mg AGe·g^−1^ for the rainy season, with no significant differences between the two seasons (*p* > 0.05). On the other hand, in the case of vegetation, V3, corresponding to *R. communis*, had a concentration of 139.61 mg AGe·g^−1^ (DW), which was the highest, as shown in Table 6.

#### 3.5.2. Total Flavonoids

The analysis of flavonoids in propolis revealed an average concentration of 5.7 ± 0.37 µg Qe·g^−1^, with P1 having the highest value of 6.13 ± 0.15 µg Qe·g^−1^, with no significant difference between seasons (*p* > 0.05), as shown in Table 7. In the case of vegetation, the highest concentration was found in V3, *R. communis* (Table 7).

#### 3.5.3. Organic Acids in Honey

The average free acidity in honey was 13.74 ± 2.33 meq OAc·kg^−1^ (FW) for the dry season and 17.68 ± 3.27 meq OAc·kg^−1^ (FW) for the rainy season, showing a significant difference between seasons (*p* < 0.05) (Table 8).

#### 3.5.4. Hydrogen Peroxide

The average value obtained for this parameter in the dry season was 3.61 ± 0.66 mg·g^−1^ (FW), while for the rainy season, the value recorded was 4.63 ± 0.85 mg·g^−1^ (FW) (Table 9); these findings were corroborated by Student’s *t* test (*p* < 0.05). The rainy season is the season with the greatest antioxidant activity.

#### 3.5.5. L-Ascorbic Acid

The average concentration of this compound in honey in the dry season was 2.90 ± 0.58 mg·g^−1^ (FW), while in the rainy season, it was 2.89 ± 0.54 mg·g^−1^ (FW), with no significant difference (*p* > 0.05). For propolis, the average concentration of this compound for the dry season was 7.97 ± 4.53 mg·g^−1^ (FW), while for the rainy season, it was 9.46 ± 5.38 mg·g^−1^ (FW), with no significant difference between seasons (*p* > 0.05) (Table 10).

#### 3.5.6. Antioxidant Capacity

The analysis of honey samples at concentrations up to 1000 ppm revealed an average AC_50_ for the dry season of 284.60 ± 29.07 µg·mL^−1^ (FW), while in the rainy season, the average CA_50_ was 239.38 ± 17.96 µg·mL^−1^, with no significant difference between seasons (*p* > 0.05). Propolis had an average CA_50_ of 412.61 ± 49.23 µg·mL^−1^ for the dry season and 344.26 ± 14.19 µg·mL^−1^ for the rainy season, indicating a significant difference between seasons (*p* < 0.05), with the rainy season presenting the greatest antioxidant activity. In the case of vegetation extracts with possible biological activity, the best CA_50_ was located in V3 (Table 11).

#### 3.5.7. Antimicrobial Analysis

In the antimicrobial analysis of the ethanol extract of propolis, the samples collected in both seasons showed activity against *S. aureus*, with no significant difference (*p* > 0.05). Regarding the analysis of the activity of these samples against *E. coli*, none of the samples collected in either season showed activity against this bacterium, as shown in Table 12. For the antifungal activity against *C. albicans*, only P2 (2020) and P6 (2021) showed activity against this yeast.

The results of the antimicrobial analyses of honey showed that all the samples analyzed that were collected in both the dry and rainy seasons presented antibacterial activity against *S. aureus*, with an average inhibition halo of 10.7 mm and no significant difference between seasons (*p* > 0.05). Regarding the activity of these samples against *E. coli*, an average inhibition zone of 10.19 mm was observed for the dry season and 11.56 mm for the rainy season, presenting a significant difference between seasons (*p* < 0.05), with the honey collected in the rainy season presenting the greatest antibacterial effect compared to that collected in the dry season.

For the plant species, the ethanolic extract of V2 (*M. citrina*) showed antibacterial activity against *S. aureus* but not against *E. coli* and no antifungal activity. V1, V3, and V4 did not exhibit antibacterial or antifungal activity.

## 4. Discussion

When comparing the results obtained in this study with those of other studies, we agree that botanical origin defines the physicochemical characteristics of honey and propolis since both vegetation and regional and climatic conditions result in a particular product [21]. These characteristics promote better acceptance and quotation of these products in the national and international markets [14,39].

### 4.1. Color

#### 4.1.1. Color in Honey

The color of honey is closely related to both the concentration of organic acids and some phenols and flavonoids through phenolic pigments such as flavanones, flavanones, and flavanols, allowing the color of honey to change due to variable concentrations of these pigments, up to 6 mg/kg of honey; therefore, it is expected that dark honey with a higher degree of acidity has greater biological activity [40]. The above could be observed in the results obtained for the honey samples studied; although there was a difference in color by season, both colors are considered dark honeys that present biological activities, such as antibacterial action on the strains studied. These results are similar to those presented by Ciappini et al. [40] and Vit et al. [41], corroborating that phenolic compounds such as flavonoids, at low concentrations, are responsible for the color of honey and that when increased, they promote dark colors. At the same time, high concentrations of phenols can be considered an indicator of antioxidant capacity, which in the case of honey allows it to have various biological activities, such as antibacterial activity.

#### 4.1.2. Propolis Color

The color of propolis, which is usually light and dark brown, is due to the incorporation of resins and waxes, which provide phenols and flavonoids such as chrysin, one of the many phenolic pigments present in up to 1000 mg/kg propolis [26]. However, as mentioned by authors such as [42,43,44], there are green propolis due to the incorporation of terpenes from different botanical species, such as *Baccharis linearis*, where the bees obtain balsams that, when incorporated into the mixture, promote green propolis; a similar process to that of the studied propolis, where the incorporation of balsams from species of the Brassicaceae family (family determined through palynological analysis) promotes a grayish green color in the samples since these balsams incorporate oleoresins, products of the oxidation of the essential oils, which in this state incorporate benzoic and cinnamic acid to the propolis, which promotes or potentiates the biological properties of this product.

### 4.2. Botanical Origin

The botanical origin of honey and propolis is a topic of interest because it allows us to define the source of resources, infer possible physicochemical characteristics, and determine the denomination of origin. Therefore, techniques such as melissopalynological analysis of honey and propolis are important in this context [14,15].

#### 4.2.1. Melissopalynological Analysis

The data obtained in the melissopalynological analysis are important for various purposes, such as when applying the classification given by Sawyer [27]. Although these honeys are classified as monofloral, it can be assumed that they include a wide variety of species that incorporate other phytochemicals different from the main pollen type, which, through a synergistic effect, potentiate the biological properties of honey [15]. At the same time, this analysis allowed us to identify species with allergenic potential, such as *R. communis* and *F. uhdei*, which, although present in traces of less than 4%, can exacerbate allergic reactions in people who are already sensitized, as shown in the research of [45], which revealed 31 different taxa of pollen types with allergenic potential.

#### 4.2.2. Obtaining Extracts

The methodology applied to propolis for pollen extraction, based on the disintegration of its components by means of solvents classified according to their polarity, not only allowed the release of pollen grains under optimal conditions for palynological analysis and adequate observation of pollen structures but also allowed the use of ethanolic and hexanolic extracts, which are useful for physicochemical analysis compared to other methodologies that perform these investigations in isolation with different sample proportions for each methodology [25].

#### 4.2.3. Wax Content of Propolis

As previously mentioned, with the aforementioned methodology, a hexane extract was obtained that degreased the sample, allowing us to obtain the waxes that constitute the propolis, which are an element in the characterization of the quality of the propolis. The percentage of waxes allowed us to designate the quality of the propolis since, as mentioned [26], the percentage of waxes should not exceed 25% of the total of the sample; in the case of the studied propolis, the percentage was 10.44% of the samples collected during the dry season and 7.24% of the samples collected in the rainy season, indicating that the propolis was of good quality and was of better quality than the propolis collected in the dry season. So, it is assumed that this result was due to the implementation of propolis collectors, which greatly reduced the incorporation of other components and increased the availability of resources [11,24,26].

#### 4.2.4. Botanical Origin of Propolis

The palynological analysis of the propolis samples from both seasons showed that it is mainly composed of pollen of the genus *Brasica*, more precisely of the species *B. rapa*, with percentages higher than 45%, as proposed by Knox’s classification; however, like honey, it incorporates a wide variety of other resources from other species. The results shown are relevant because although various investigations [42,43] have shown the percentage of balsam incorporation, it has not been clear since it is mostly assumed to be a base of different resins from various tree species, as mentioned [42,43]. At the same time, and as in honey, it was possible to observe the incorporation of resources from a wide variety of species, such as those observed in honey, which would also be by means of a synergistic act potentiating the biological properties of propolis. However, this analysis, similar to that for honey, allowed the elucidation of species with allergenic potential, which, although they are considered to be traces, could increase the percentage of the integrated resource that could exacerbate the allergic reaction of already sensitized individuals.

### 4.3. Floral Calendar

With the information obtained from the palynological analysis and the determination of the botanical species around the hive, a flowering calendar was established that allowed us to observe how the sources of resources are more abundant in the period from June to November (rainy season) than in the period from December to May (dry season). A wide variety of species of the *Brassica* genus and the Asteraceae family have been established as stand plants, and these plants are known to be diverse resources for beehives, such as nectar, pollen, and balsams, the latter of which are relevant for the development of propolis [3,37,38,46,47,48,49].

### 4.4. Total Phenols

#### 4.4.1. Total Phenols

The phenol concentration in honey and propolis is a parameter that allows the quality level to be determined, since these components promote the physical and chemical properties of honey and propolis, in such a way, that both international and national regulations designate that both honey and propolis of better quality are those that present a higher concentration of these compounds [14,24,39].

#### 4.4.2. Phenols in Honey

The physical characteristics of honey, such as color, odor, and flavor, are closely related to its phenolic profile; in turn, these compounds are responsible for the antioxidant activity of honey, so knowledge of these compounds is key to understanding the chemistry of honey, and their understanding may allow this to become a quality parameter [14]. In honey, phenolic compounds account for up to 6000 μg·kg^−1^ of honey, with flavanones, flavonones, and flavonols being the most recognized; these compounds have been identified as components of nectar and pollen [14,40]. In the honey samples studied, phenol concentrations were similar between both seasons and did not differ significantly. In particular, in 2020 (M3 and M4), a 23% increase in the concentrations of these compounds was observed compared to that in 2019 (M1 and M2), and palynological analysis did not reveal an increase in the number of honey flora species [40]. This result can be explained to some extent by the ecological restoration caused by the paralysis of daily, economic, and industrial life during the SARS-CoV-2 pandemic, which allowed an increase in the populations of different resource species, increasing the availability of certain resources that, in another context, would be scarce or nonexistent [40].

#### 4.4.3. Total Phenols in Propolis

The phenols present in propolis are components that promote some of its properties, such as phenolic pigments that promote the characteristic color of propolis, representing approximately 1000 mg kg^−1^ [50]. Some of the components that are usually present are benzoic acid, which, with other substituents, forms acids such as phenolic, caffeic, ferulic, coumaric, and cinnamic acids that contribute to the bactericidal and fungicidal properties of propolis [50]. In the propolis samples studied, the concentration of phenols did not differ significantly between seasons; however, an increase was observed at the end of 2020 at P4, with a concentration of 142.56 mg GAe·g^−1^, corresponding to the rainy season harvest, which, as in honey, is a consequence of a greater availability of resources due to sanitary confinement [40,50,51]. On the other hand, the data obtained here in comparison with the results of [15] are more conservative due to parameters such as the collection method, which, in the case of this research, the use of propolis collectors (indicated in the official standards [24]) and the programming of harvesting times (collection effort) allows a better representation of the composition of propolis, which cannot be corroborated in the aforementioned study.

#### 4.4.4. Plant Phenols

The results obtained for this item showed that the contribution of phenols is possibly not necessarily from the species with the highest concentration but from the species with the highest abundance, which is usually the most preferred by the bees. In this study, the phenolic load of the species of the *Brassica* genus, such as the species *B. rapa*, was lower than that of *M. citrina*. It should be noted that the participation of the phenolic load contributed by the other species is not negligible, but, on the contrary, through a synergistic act, together with the enzymatic addition, given the contribution of saliva to the nectar or to the mixture of protopropolymer, provides the necessary requirements to physically and chemically restructure both honey and propolis, promoting a suitable means to preserve and form a new phenolic index for each production [14,50,51].

### 4.5. Total Flavonoids

#### 4.5.1. Flavonoids in Honey

The concentration of flavonoids in honey is derived from the concentration of phenolic compounds such as benzopyran, which, depending on oxidation, can be in the form of flavones, flavonols, flavonones, isoflavones, and flavanes [14,50]. However, the content of flavonoids in honey is minimal enough to indicate representative biological activity, and the biological properties are attributed to other compounds that work in synergy; therefore, this parameter was not considered for honey in the present investigation [14,51].

#### 4.5.2. Propolis Flavonoids

The flavonoids present in propolis are derived from esters and phenolic acids, while the concentration and type of flavonoids depend on the propolis vegetation, season, and region of production [52,53,54]. However, in the present study, it was not possible to determine a significant difference between seasons due to the homogeneity of the surrounding flora, which does not present considerable variations in terms of diversity but rather in terms of disposition, which, depending on climatic factors or agricultural work carried out in the study area, may favor the increase or increase of populations, allowing a greater disposition of resources such as *B. rapa* [3,50,51]. In comparison with other studies carried out on propolis in the area, the data obtained here are greater than the data obtained by [22] due to the collection methods, collection effort, processing, and storage methodologies. 

#### 4.5.3. Plant Flavonoids

Regarding the concentration of flavonoids in the plants studied, V1 (*B. rapa*) with 2.96 µg Qe·g^−1^ and V2 (*M. citrina*) with 1.96 µg Qe·g^−1^ presented concentrations that could represent an important contribution to the concentration of flavonoids in the propolis, which contained an average of 5.7 µg Qe·g^−1^. The opposite trend occurs for V3 (*R. comunnis*) at 5.43 µg Qe·g^−1^ and V4 (*F. uhdei*) at 0.87 µg Qe·g^−1^. Although V3 has a concentration similar to that of the propolis, in the palynological analysis, the presence of this compound is low, at only 3%, and thus, its contribution is minimal, similar to that of V4 but with a lower concentration [48,52].

### 4.6. Acidity

#### 4.6.1. Acidity

In propolis, as in honey, the concentration of free acids (organic acids) is dependent on the botanical origin [3,14]. In honey, free acidity promotes color, aroma, and flavor, as well as chemical properties such as electrical conductivity and pH. It is also a quality reference since concentrations higher than 40 meq OAc·g^−1^ indicate that the honey is undergoing fermentation due to the enzymatic reactions occurring during honey maturation and storage [55,56,57,58,59,60,61,62]. Therefore, the honey studied here is considered to be of good quality since it does not exceed the established limit, having a minimum concentration of 10.90 meq OAc·kg^−1^ and a maximum concentration of 22.17 meq OAc·kg^−1^. Notably, high concentrations were detected in the honeys harvested during the rainy season, with the 2020 harvest (M4) having the highest concentration of free acids.

#### 4.6.2. Ascorbic Acid Concentration in Honey

L-ascorbic acid, also called vitamin C, hexuronic acid, or antiscorbutic factor, is considered an excellent antioxidant that reduces oxidation by reacting with the superoxide ion O_2_^−^ and singlet oxygen (atomic) as HOO^−^ or OH^−^ by dehydrogenation to generate dehydroascorbate [61]. In honey, this acid is present at a low concentration, generating conflicting opinions about its participation in antioxidant activity since in vitro evaluations have shown that it possesses antioxidant activity only at concentrations higher than those present in honey (0.5 to 2.5 mg/100 g); therefore, some authors comment that such a concentration of L-ascorbic acid does not participate in the antioxidant activity of honey [61]. However, some honey, such as Manuka honey, which has a concentration of 1.067 mg·g, has excellent antioxidant activity, and thus, concentrations greater than 1 mg·g^−1^ of L-ascorbic acid, which generally promotes a dark color in honey, are indicative of greater antioxidant activity than light-colored honey, which usually has a low concentration of L-ascorbic acid. Based on the results obtained here, although a difference was observed between light amber honey collected in the dry season and amber honey collected in the rainy season, both honey samples were in the range of dark honey for NOM-004-SAG/GAN-2018 [39], and on the Pfund scale, the concentrations of L-ascorbic acid within the range established for honey (0.5 to 2.5 mg/100 g) were obtained for both seasons. The average concentration was 2.91 mg·g for the rainy season and 2.89 mg·g^−1^ for the dry season, with no significant difference between seasons.

#### 4.6.3. Hydrogen Peroxide

At the same time, the products obtained from the formation of organic acids have received great interest. Although they do not exceed 0.5% of the honey volume, these products have a wide range of antibacterial properties since compounds such as hydrogen peroxide, the main antibacterial agent, previously called inhibin, are compounds formed from the production of glycolic acid [56,57,59,63,64]. Therefore, in the samples studied here, the concentration of this component was greater in the honey collected in the rainy season, at 5.78 mg·g^−1^, than in the honey collected in the dry season, in which a maximum concentration of 4.36 mg·g^−1^ of honey was obtained. This finding is significant since the formation and concentration of hydrogen peroxide, as well as other components, are closely related to the botanical origin, quality, and constituents of the nectars used by honeybees. Compared with the botanical use obtained through palynological analysis, nectar sources are more abundant in the rainy season since during this season, the plants are more developed and exhibit an increase in diversity [3].

#### 4.6.4. Exploitation of Hydrogen Peroxide

In turn, as previously mentioned, the hydrogen peroxide contained in honey is an agent that provides honey with antibacterial properties [12]; this relationship can be observed by comparing the average concentration of hydrogen peroxide obtained in each of the seasons against the diameter of inhibition in the analysis of the antibacterial activity against *E. coli*. A directly proportional relationship was observed, in which a higher concentration of hydrogen peroxide reflects greater antibacterial activity, with the average concentration of this compound in the dry season being 3.62 mg·g^−1^, resulting in an average inhibition halo of 10.20 mm, compared with that obtained in the rainy season, where a higher concentration of 4.63 mg·g^−1^ promoted an inhibition halo of 11.56 mm, with a significance value of 0.04 between seasons [19]. However, this relationship was not appreciable when we compared the data obtained with the same concentration for the inhibition diameters obtained for the *S. aureus* strain, which had an inhibition diameter of 10.26 mm for the sample collected in the dry season at a concentration of 3.62 mg·g^−1^, while an inhibition diameter of 10.05 mm was obtained for the sample collected in the rainy season with a concentration of 4.63 mg·g^−1^ [19]. No significant difference was observed between the antibacterial activities of honey collected in both seasons since such activity is related not only to hydrogen peroxide but also to other phytochemical compounds present in honey [19].

#### 4.6.5. Nonperoxide Antibacterial Activity

This type of activity occurs because the antibacterial property of honey depends not only on hydrogen peroxide but also on other agents, such as organic acids, aromatics, caffeic acid, ferulic acid, 2-hydroxyphenylpropionic acid, and 1,4-dihydroxybenzene, which, together with a low pH and high osmolarity, promote the so-called nonperoxide antibacterial activity (NPAA) in honey generated by the phytochemical components provided by the botanical sources of origin. Therefore, several studies have attributed this capacity to the presence of methylglyoxal; so, it is suggested in future research to perform relevant evaluations to corroborate the presence of this compound [3,19,61,62].

### 4.7. Antioxidant Capacity

#### 4.7.1. Antioxidant Capacity of Honey

For the antioxidant capacity, the honey samples were characterized by a greater AC_50_ than the control (quercetin); however, as there was no significant difference between seasons and as could be observed in the antibacterial activity, a difference in the action of this property is not expected. This finding coincides with the results of Ciappini and collaborators [40], who concluded that the higher the concentration of phenols is, the better the AC_50_, and because of this, the AC_50_ of honey will always be different from 0, so it is expected to present some of its properties [3,14,65]. This equality in the honey studied is due to the development of arvense flora between seasons due to irrigation induced by agricultural activity, which favors the out-of-season growth of species from different families, as observed in the flowering calendar, promoting the acquisition of a wide range of phenolic compounds [3].

#### 4.7.2. Antioxidant Capacity of Propolis

The antioxidant capacity of propolis is mainly determined by the phenolic compounds that constitute it as a result of various actions, such as glycolysis caused by enzymes present in bee saliva, as well as the inclusion of other constituents such as balsams or pollen, or by other phytochemicals that allow propolis to acquire antioxidant, antibacterial, antifungal, and antiviral properties [20]. In the present study, the antioxidant capacity of propolis was greater than that of the quercetin control, with higher concentrations than those obtained in previous studies, possibly due to the use of propolis collectors and the collection effort that allowed a better representation of the concentrations of this product [22,26,47].

### 4.8. Antimicrobial Activity

#### 4.8.1. Honey Antibacterial Activity

The antimicrobial activity of honey is widely recognized since it influences both Gram (−) and Gram (+) bacteria through the action of hydrogen peroxide or through the influence of phytochemical components provided by the honey flora, which are known to have antibacterial effects unrelated to hydrogen peroxide [3,64,66,67,68]. Both effects were present in the antibacterial action of the honey samples against the strains studied since the inhibitory action against Gram (+) *S. aureus* bacteria is mainly inhibited by the action of hydrogen peroxide, while the inhibitory action against Gram (−) *E. coli* bacteria involves the joint action of hydrogen peroxide plus the nonperoxide effect involving phenolic compounds (flavonoids and phenolic acids), nitrogen compounds, vitamins, pH, and low humidity, which can even have an effect on strains with antibiotic resistance. These results and reasoning are similar to those presented by Estrada and collaborators [64], who obtained similar results and suggested that hydrogen peroxide is a possible inhibitory agent [64,69,70,71].

#### 4.8.2. Antifungal Activity

The mode of action of honey against pathogenic fungi is not well known, but some researchers have argued that its antifungal activity is similar to its nonperoxide antibacterial activity and therefore depends on the physicochemical components of the existing flora [3,72]. On the other hand, the honey samples studied, classified as monofloral honey of wild turnip, presented results similar to those of other studies on honeys of this type; more specifically to the works with honey of *B. rapa*, where researchers have determined that this type of honey presents low or no antifungal activity, which in contrast to its antibacterial activity, properties such as acidity and high sugar content, do not exert any inhibitory effect on fungi and yeasts. In contrast, instead of promoting antibacterial activity, these properties could favor the growth of these organisms [72].

#### 4.8.3. Propolis Antibacterial Activity

Regarding the antibacterial activity of propolis, several investigations have shown that, depending on its phytochemical composition, it may or may not present antibacterial activity against Gram (−) bacteria since, unlike honey, propolis does not incorporate hydrogen peroxide in its constitution; so, its antibiotic properties depend on compounds such as phenols and flavonoids [64,70]. Fourteen flavonoids have been identified, among which quercetin, apigenin, and 3,6,7,3,4-pentahydroxyflavone are involved in DNA replication and can inhibit the DNA gyrase enzyme involved in DNA replication in *E. coli* [73]. However, the results obtained showed that the propolis collected in Cuautitlán did not show such activity, which could be related to the concentrations of phenols such as flavonoids, which might not be sufficient to inhibit the growth and development of *E. coli*. However, the fact that the propolis studied did not have inhibitory effects on this bacterium should not be an argument against its antibacterial activity, since propolis acts in synergy with various antibiotics, such as amoxicillin and cephalexin, to alter the permeability of the internal and external membranes. It should also be noted that the inhibitory action of propolis occurs between 24 and 48 h, which may be one of the reasons why no activity was observed in the present study, since only 24 h was considered, and it is also possible that the propolis studied lacks the phytochemicals responsible for such activity [3,73,74].

#### 4.8.4. Antifungal Activity of Propolis

In terms of antifungal activity, propolis has an inhibitory effect on *C. albicans* strains due to the action of compounds such as caffeic acid, benzyl p-coumaric acid, pinocembrin, and pinobanksin, which inhibit mycelial growth by affecting respiration and energy homeostasis. However, as observed in the results obtained when the inhibitory action is weak or null, propolis can act synergistically with antifungal agents and enhance their potential [75,76].

#### 4.8.5. Antimicrobial Activity of Plant Extracts

Antimicrobial analysis of the ethanolic extracts of the plants with potential biological properties revealed antibacterial activity only against the *S. aureus* strain of the species *M. citrina* (V2). Although the other species did not show antibacterial or antifungal activity, it should be considered that the phytochemicals, together with the biological components provided by the bees, such as saliva and wax, may complement each other in a synergistic manner, forming new components that promote various biological actions [3,14,26].

## 5. Conclusions

This study highlights the importance of utilizing resources such as ruderal vegetation, which can result in the consistent production of high-quality honey and propolis with commercial, nutritional, and medicinal value. The diversity and availability of melliferous and propoliferous flora each season influence the physical and chemical properties of honey and propolis. Palynological analyses allow for the determination of botanical origin, which is essential for understanding these characteristics. Determining the botanical origin and analyzing the physical and chemical characteristics of both products promote better utilization of the resources surrounding the hive, including species considered invasive in crops. For example, *B. rapa* becomes significant as the use of these melliferous, polliniferous, and propolis resources generates consistent production of honey and propolis with optimal quality and beneficial properties for commercial, nutritional, and medicinal purposes.

Additionally, while the phytochemical contribution is derived from the melliferous flora, it should not be inferred that species with higher pollen percentages are solely responsible for the biological properties of honey and propolis. As observed in this study, species such as *B. rapa*, the main pollen type, did not exhibit high concentrations of phenols or flavonoids or antibacterial activity. Conversely, species such as *M. citrina*, which showed antibacterial activity and high phenol concentrations, were represented to a lesser extent in honey and propolis. Therefore, these products should be considered synergistic conglomerates, where the addition of enzymes and lipids by bees, along with other phytochemicals from minor species, provides the ideal conditions for generating the various biological actions of honey and propolis.

The analyses and methodologies proposed in this study promote better market acceptance by demonstrating the antioxidant and antibacterial effects of honey and propolis, as well as their potential adverse effects, such as their allergenic potential, due to the inclusion of allergens from nectar, pollen, and resins. In this study, the allergens were primarily from *F. uhdei* and *R. communis*. Hence, consumer warnings can mitigate the risk of exacerbated allergic reactions in individuals already sensitized to these species.

## Figures and Tables

**Table 1:** Analysis of honey samples from the FES-C group.

	M1	M3	M5	M7	M2	M4	M6	M8
Color mm	84	83	80	86	111	97	114	105
	Light amber	Light amber	Light amber	Amber	Amber	Amber	Amber	Amber
Brix %	80.6	80.0	80.4	82.1	83.8	82.5	87.0	82.0
Fructose %	56.7	53.1	56.2	52.8	56.9	51.6	56.7	53.12
Moisture	19.1	19.1	19.7	19.4	19.4	19.7	19.2	19.0
pH	3.42	3.53	3.58	3.14	4.22	3.75	3.26	4.17

M, 1, 3, 5, 7 honey dry season; M, 2, 4, 6, 8 honey rainy season.

**Table 2:** Yields of extracts obtained from propolis collected during both dry and rainy seasons.

Extract Yields			
		Methanol Extract %	Hexanic Extract %
Rainy season	P1	35.49	7.00
	P3	23.30	6.19
	P5	49.27	8.86
	P7	21.39	6.91
Dry season	P2	24.13	10.07
	P4	25.21	11.47
	P6	22.70	10.56
	P8	17.27	9.67

P1, 3, 5, 7, Propolis rainy season; P2, 4, 6, 8, Propolis dry season.

**Table 3:** The palynological spectrum of honey samples from FES-C; the numbers of families, genera, and species are shown in blue, and the pollen percentage of the predominant pollen is shown in green.

	Dry Season				Rainy Season			
	M1	M3	M5	M7	M2	M4	M6	M8
Family	15	15	15	14	19	20	18	19
Genus	24	23	30	20	34	25	22	33
Species	24	22	29	17	33	29	35	32
*Amaranthus hybridus* L.					2.08	1.33	1.58	
*Ambrocia psilostachya* DC.					1.33			
*Ambrosia psilostachya* DC.		2.08						1.92
*Bidens*	2.83	4.58	3.00	7.00	9.42	1.00	9.17	9.00
*Brassica*	47.83	70.75	59.00	51.67	39.75	40.67	39.17	44.92
*Casuarina equisetifolia* L.						1.33	1.00	
*Eucayptus camaldulensis* Dehndhardt		4.92	4.58	3.00		5.00	2.67	4.25
*Ficus*			2.25		1.92	2.67		
*Fraxinus uhdei* (Wenz.) Ligelsh		3.42	3.83	4.33			2.83	2.08
*Helminthotheta echioides* (L.) Holub	1.58					2.33	2.67	
*Ligustrum japonicum* Thumb			1.05		2.50			
*Medicago Sativa* L.				1.00				
*Melaleuca citrina* (Curtis) Dum. Cours	31.67		8.08	10.00	22.00	23.67	22.00	18.58
*Nicotiana glauca* Graham			2.50					
*Parthenium hysterophorus* L.		1.00	1.42	4.33				
*Pronus*					1.08		2.33	
*Ricinus communis* L.	3.42	2.75	2.92	5.00	3.00	3.67	2.08	4.33
*Rumex* sp.						1.00		
*Salix*	1.00			2.67		1.67	1.67	2.08
*Schinus molle* L.	5.58	1.58	1.75	7.00	8.67	9.00	8.25	4.75
*Solanum elaeagnifolium* Cav.	1.25		2.00	1.67	2.08			
*Solanum elaeagnifolium* Cav.						1.33	1.25	
*Solanum rostratum* Dunal.					1.58			1.08
*Sphaeralcea angustifolia* (Cav.) G. Don				1.00				
*Taraxacum campylodes* G.E.Haglund			2.92		1.83	2.33		
*Tithonia tubaeformis* (Jacq.) Cass.		1.17						
*Trifolium amabile* Kunth		3.17	3.67					
Complementary	4.83	4.58	1.03	1.33	2.75	3.00	3.33	7.00

M, 1, 3, 5, 7 honey dry season; M, 2, 4, 6, 8 honey rainy season.

**Table 4:** The pollen spectrum of the pollen contained in propolis. Samples collected in both the dry season and the rainy season are shown, with the numbers of families, genera and species shown in blue and the percentage of the main pollen type shown in green.

	Dry Season				Rainy Season			
	P1	P3	P5	P7	P2	P4	P6	P8
Family	27	19	18	18	19	20	19	16
Genus	30	27	23	22	26	24	25	20
Species	18	15	12	11	12	11	12	12
*Acacia retinodes* Schltdl	2.00	2.25			2.83	3.12	3.75	2.61
*Alnus*	1.76	1.75	2.00	1.92	3.42	2.65		4.39
*Bidens*			4.17	3.58				2.91
*Brassica*	63.08	55.17	52.50	50.42	52.17	63.25	43.33	55.58
*Bursera*							3.25	
*Casuarina equisetifolia* L.	1.75	1.75					2.17	
*Cupresus lusitanica* Mill	3.17	4.83	2.42	3.75	4.75	4.74		7.00
*Eucayptus camaldulensis* Dehndhardt	4.83	4.50	7.17	6.00	4.83	4.33	8.50	3.75
*Ficus*								
*Fraxinus uhdei* (Wenz) Lingelsh	4.00	4.17		3.00	4.00	4.00	5.00	3.92
*Helninthotheta echioides* (L.) Holub		4.17		1.67			4.75	
*Ipomea purpurea* L. Roth					1.42			
*Ligustrum japonicum* Thumb				3.00	4.92	3.25	1.92	2.92
*Melaleuca citrina* (Curtis) Dum. Cours	5.00		11.25	8.83	8.75	5.00	10.92	1.67
*Nicotiana glauca* Graham				1.00				
*Pinus*		1.17			3.75	2.10	1.33	4.42
*Populus*			2.75	1.92	1.33		2.75	
*Pronus*		5.49	5.74	1.33				
*Ricinus communis* L.	2.83	5.75	4.92	7.00	2.83	1.08	2.51	2.67
*Schinus molle* L.	1.08	1.38			1.08	1	1.40	2.58
*Taraxacum campylodes* G.E.Haglund								3.00
*Trifolium amabile* Kunth	3.75	3.13	1.92	1.83			3.50	
Complementary	6.75	4.50	5.17	4.75	3.92	5.48	4.92	2.58

**Table 5:** Flowering calendar of species surrounding the hive that serve as a source of resources such as nectar, pollen, resins, and balsams.

Honey	Prop	Family	Genus	Species	Dry Season									Rainy Season									
					December	January	February	March		April		May		June		July		August		September		October	November
		Altingiaceae	*Liquidambar*	*Liquidambar styraciflua* L. var. mexicana Oerst																			
		Amaranthacaeae	*Amaranthus*	*Amaranthus hybridus* L.																			
		Anacardiaceae	*Schinus*	*Schinus molle* L.																			
		Asparagaceae	*Yucca*	*Yucca elephantipes* Rangel.																			
		Asteraceae	*Ambrocia*	*Ambrosia psilostachya DC.*																			
			*Baccharis*	*Baccharis salicina* Tor. & A. Gray																			
			*Bidens*	*Bidens aurea* (Aiton) Sherff																			
				*Bidens odorata* Cav.																			
				*Bidens pilosa* L.																			
			*Galinsoga*	*Galinsoga parviflora* Cav.																			
			*Helminthotheta*	*Helminthotheta echioides* (L.) Holub																			
			*Parthenium*	*Parthenium hysterophorus* L.																			
			*Simsia*	*Simsia amplexicaulis* (Cav.) Pers.																			
			*Taraxacum*	*Taraxacum campylodes* G.E.Haglund																			
			*Titonia*	*Tithonia tubaeformis* (Jacq.) Cass.																			
			*Viguiera*	*Viguiera excelsa* (Willd.) Benth. & Hook. F.																			
		Brassicaceae	*Brassica*	*Brassica rapa* L.																			
			*Raphanus*	*Raphanus raphanistrum* L.																			
			*Sisymbrium*	*Sisymbrium irio* L.																			
		Burseraceae	*Bursera*																				
		Casuarinaceae	*Casuarina*	*Casuarina equisetifolia* L.																			
		Convolvulaceae	*Ipomoea*	*Ipomoea purpurea* L.																			
		Cucurbitaceae	*Sicyus*	*Sicyos deppei* G. Don																			
		Cupressaceae	*Cupressus*	*Cupressus lusitanica* Millere																			
		Euphorbiaceae	*Ricinus*	*Ricinus communis* L.																			
		Fabaceae	*Acacia*	*Acacia retinodes* Schtdl.																			
			*Medicago*	*Medicago Sativa* L.																			
			*Trifolium*	*Trifolium amabile* Kunth																			
		Malvaceae	*Sphaeralcea*	*Sphaeralcea angustifolia* (Cav.) G. Don																			
		Moraceae	*ficus*	*Ficus retusa* L.																			
		Myrtaceae	*Eucalyptus*	*Eucalyptus globolus* Lábill																			
			*Melaleuca*	*Melaleuca citrina* (Curtis) Dum. Cours																			
		Olaceae	*Fraxinus*	*Fraxinus uhdei* (Wenz.) Ligelsh																			
			*Ligustrum*	*Ligustrum japonicum* Thunb.																			
		Onagraceae	*Oenothera*	*Oenothera elata* Kunth																			
				Oenothera rosea L’Hér. ex Aiton																			
		Pinaceae	*Pinus*																				
		Poaceae	*Zea*	*Zea mays* L.																			
		Polemoniaceae	*Loeselia*	*Loeselia mexicana*																			
			*Rumex*	*Rumex* sp.																			
		Polygonaceae	*Persicaria*	*Persicaria hydropiperoides* (Michx.) Small																			
				*Persicaria Lápathifolia* (L.) Delarbre																			
		Resedaceae	*Reseda*	*Reseda luteola* L.																			
		Rosaceae	*Pronus*																				
			*Rosa*	*Rosa canina* L.																			
		Salicaceae	*Populus*																				
			*Salix*	*Saix babylonica* L.																			
		Solanaceae	*Jaltomata*	*Jaltomata procumbens* (Cav.) J. L. gentry																			
			*Nicotiana*	*Nicotiana glauca Graham*																			
			*Solanum*	*Solanum elaeagnifolium* Cav.																			
				*Solanum rostratum* Dunal																			
														Honey sorce					Flowering period of the main pollen type				
														Propolis source					Flowering period of secondary pollen				
														Dry harvest period					Allergenic pollen flowering period				
														Rainy harvest period					Peak nectar production				
														Flowering period					Peak pollen production				

**Table 6:** Concentrations of total phenols in propolis, honey, and plant species (mg AGe·g^−1^).

Dry Season		Rainy Season	
M1	1.78	M2	1.85
M3	2.20	M4	2.29
M5	1.76	M6	1.87
M7	1.65	M8	1.56
P1	62.79	P2	65.00
P3	29.51	P4	142.56
P5	61.86	P6	57.70
P7	58.98	P8	53.00
V1	39.78	V3	139.61
V2	104.30	V4	70.86

M, 1, 3, 5, 7 honey dry season; M, 2, 4, 6, 8 honey rainy season; P, 1, 3, 5, 7 propolis dry season; P, 2, 4, 6, 8 propolis rainy season; V1, *B. rapa*; V2, *M. citrina*; V3, *R. communis*; V4, *F. uhdei*.

**Table 7:** Concentrations of flavonoids in propolis and plant species with various biological properties (µg Qe·g^−1^).

Dry Season		Rainy Season	
P1	6.13	P2	5.86
P3	5.99	P4	5.50
P5	5.28	P6	5.78
P7	5.77	P8	5.74
V1	2.96	V3	5.43
V2	1.96	V4	0.87

P, 1, 3, 5, 7 propolis dry season; P, 2, 4, 6, 8 propolis rainy season; V1, *B. rapa*; V2, *M. citrina*; V3, *R. communis*; V4, *F. uhdei*.

**Table 8:** Free acidity values (meq OAc·kg^−1^) of the Cuautitlan honey collected in both seasons.

Dry Season		Rainy Season	
M1	10.90	M2	14.47
M3	16.30	M4	22.17
M5	13.50	M6	17.70
M7	14.27	M8	16.40

M, 1, 3, 5, 7, honey dry season; M, 2, 4, 6, 8, honey rainy season.

**Table 9:** Hydrogen peroxide analysis of Cuautitlan honey collected over two seasons (mg/g).

Dry Season		Rainy Season	
M1	2.76	M2	3.76
M3	4.36	M4	5.78
M5	3.58	M6	4.71
M7	3.76	M8	4.27

M, 1, 3, 5, 7, honey dry season; M, 2, 4, 6, 8, honey rainy season.

**Table 10:** L-ascorbic acid concentrations in propolis and honey (mg/g) collected during the seasons of 2019–2022 (dry and rainy).

Dry Season		Rainy Season	
M1	2.73	M2	3.68
M3	3.63	M4	2.43
M5	3.03	M6	2.65
M7	2.24	M8	2.81
P1	5.16	P2	3.03
P3	4.15	P4	9.71
P5	14.01	P6	16.19
P7	5.78	P8	8.94

M, 1, 3, 5, 7 honey dry season; M, 2, 4, 6, 8 honey rainy season; P, 1, 3, 5, 7 propolis dry season; P, 2, 4, 6, 8 propolis rainy season.

**Table 11:** The AC_50_ values (µg·mL^−1^) of propolis, honey and plant species with biomedical properties are presented. As a control, the value for quercetin (Q) is presented.

Dry Season		Rainy Season	
M1	297.51	M2	239.37
M3	246.48	M4	256.14
M5	279.91	M6	247.55
M7	314.51	M8	214.46
P1	486.43	P2	357.56
P3	389.43	P4	346.2
P5	388.27	P6	324.24
P7	386.31	P8	349.07
V1	-	V3	41.21
V2	8.40	V4	2320.56
Q	3.27		

M 1, 3, 5, 7 honey dry season; M 2, 4, 6, 8 honey rainy season; P 1, 3, 5, 7 propolis dry season; P 2, 4, 6, 8 propolis rainy season V1: *B. rapa*; V2: *M. citrina*; V3: *R. communis*; V4: *F. uhdei*; Q quercetin.

**Table 12:** Antimicrobial analyses of propolis, honey, and vegetable samples collected from April and November 2019 to 2022 were performed at a concentration of 4 mg; 25 µg chloramphenicol was used as a positive control for bacteria, and 25 µg nystatin was used for yeast fungi. The diameter of inhibition is reported in mm, and n/a is listed where no activity was present.

Dry Season				Rainy Season			
	*S. aureus*	*E. coli*	*C. albicans*		*S. aureus*	*E. coli*	*C. albicans*
C+	21.74 ± 0.92	24.08 ± 0.16	15.18 ± 0.50				
P1	8.71 ± 0.25	n/a	n/a	P2	8.95 ± 0.58	n/a	8.21 ± 0.90
P3	9.85 ± 0.69	n/a	n/a	P4	9.25 ± 0.49	n/a	-
P5	8.15 ± 0.06	n/a	n/a	P6	9.48 ± 0.19	n/a	7.61 ± 0.59
P7	8.18 ± 0.04	n/a	n/a	P8	9.28 ± 0.19	n/a	-
C+	5.82 ± 0.41	9.81 ± 0.70	3.81 ± 0.41				
M1	10.31 ± 0.13	11.27 ± 0.94	n/a	M2	9.25 ± 0.54	12.35 ± 0.29	n/a
M3	9.78 ± 0.93	9.57 ± 0.52	n/a	M4	10.65 ± 0.67	10.39 ± 0.64	n/a
M5	10.22 ± 0.16	9.54 ± 0.86	n/a	M6	11.80 ± 0.55	12.07 ± 0.30	n/a
M7	10.75 ± 0.11	10.40 ± 0.10	n/a	M8	8.50 ± 0.41	11.43 ± 0.39	n/a
V1	n/a	n/a	n/a				
V2	10.42 ± 0.63	n/a	n/a				
V3	n/a	n/a	n/a				
V4	n/a	n/a	n/a				

C+: positive control; n/a: no activity; M 1, 3, 5, 7 honey dry season; M 2, 4, 6, 8 honey rainy season; P 1, 3, 5, 7 propolis dry season; P 2, 4, 6, 8 propolis rainy season V1: *B. rapa*; V2: *M. citrina*; V3: *R. communis*; V4: *F. uhdei*.

## Data Availability

Data are contained within the article.

## References

[1] Kibru G., Tassew H. (2013). Multidrug-resistant bacterial isolates in infected wounds at Jimma University Specialized Hospital, Ethiopia. Ann. Clin. Microbiol. Antimicrob..

[2] Melake N.A., Eissa N.A., Keshk T.F., Sleem A.S. (2015). Prevalence of multidrug-resistant bacteria isolated from patients with burn infection. Menoufia Med. J.

[3] Alcivar-Saldaña J.J., Rodriguez-Monroy M.A., Canales-Martinez M.M. (2023). Current Knowledge of the Melliferous Florae in Mexico Using Methodologies to Understand Bee–Plant–Human Interactions. Appl. Sci..

[4] Ball D.W. (2007). The Chemical Composition of Honey. J. Chem. Educ..

[5] Santos-Buelga C., González-Paramás A.M., Alvarez-Suarez J. (2017). Chemical Composition of Honey. Bee Products—Chemical and Biological Properties.

[6] Wali A.F., Pillai J.R., Razmpoor M., Jabnoun S., Akbar I., Rasool S., Arafah A., Khan A., Akhter R., Majid S., Rehman M.U., Majid S. (2020). Honey and Its Phyto-Constituents from Chemistry to Medicine. Therapeutic Applications of Honey and Its Phytochemicals.

[7] Bankova V.S., de Castro S.L., Marcucci M.C. (2000). Propolis: Recent advances in chemistry and plant origin. Apidologie.

[8] Peña R.C. (2008). Estandarización en propóleos: Antecedentes químicos y biológicos. Cienc. E Investig. Agrar..

[9] Lozina L., Peichoto M.E., Acosta O., Granero G.E. (2010). Estandarización y Caracterización organoléptica y físico-química de 15 Propóleos Argentinos. Lat. Am. J. Pharm..

[10] Vargas Sánchez R.D., Torrescano G.R., Sánchez A. (2013). El propóleos: Conservador potencial para la industria alimentaria. Interciencia.

[11] Cheng A.X., Han X.J., Wu Y.F., Lou H.X. (2014). The Function and Catalysis of 2-Oxoglutarate-Dependent Oxygenases Involved in Plant Flavonoid Biosynthesis. Int. J. Mol. Sci..

[12] Salamanca G. (2017). Origen, Naturaleza, Propiedades Fisicoquímicas y Valor Terapéutico del Propóleo.

[13] Santos-Buelga C., González-Paramás A.M., Alvarez-Suarez J. (2017). Phenolic Composition of Propolis. Bee Products—Chemical and Biological Properties.

[14] Muñoz O., Copaja S., Speisky H., Peña R., Montenegro G. (2007). Content of flavonoids and phenolic compounds in chilean hoeys. Quim. Nova.

[15] Louveaux J., Maurizio A., Vorwohl G. (1978). Methods of melisopalynology. Bee World.

[16] Bankova V., Popova M., Bogdanov S., Sabatini A.G. (2002). Chemical Composition of European Propolis: Expected and Unexpected Results. Z. Naturforsch. C J. Biosci..

[17] Morales J., Landa M., García S.G., Lavoignet M. (2019). Intelligent harvesting techniques for bee products applied in beehives in the Misantla region. Rev. Ingeniantes.

[18] Soto L., Elizarrarás-Baena R., Soto-Muciño I. (2017). Beekeeping situation in Mexico and perspective of honey production in the State of Veracruz. Rev. Estrateg. Desarro. Empres..

[19] García M.F., Armenteros E., García J.A., Mendez J., Ramos G. (2022). Chemical composition of the bee honey and its relation to health benefits. Rev. Med. Electrón..

[20] Chan Chi J.R., Caamal I., Pat V.G., Martínez L.D., Pérez A. (2018). Social and economic characterization of bee honey production in the north of the state of Campeche, Mexico. Textual Anal. Medio Rural. Latinoam..

[21] Campos M., Leyva C., Ferráez M., Sánchez Y. (2018). El mercado internacional de la miel de abeja y la competitividad de México. Rev. Econ..

[22] Rodríguez B., Canales M.M., Penieres J.G., Cruz T.A. (2020). Chemical composition, antioxidant properties and antimicrobial activity of Mexican propolis. Acta Univ..

[23] Mato I., Huidobro J.F., Simal-Lozano J., Sancho M.T. (2003). Significance of nonaromatic organic acids in honey. J. Food Prot..

[24] (2017). Norma Oficial Mexicana NOM-003-SAG/GAN-2017. Propolis, Production and Processing Specifications, Diario Oficial de la Federación, Diario Oficial de la Federación: Ciudad de México, Mexico. https://www.dof.gob.mx/normasOficiales/6794/sagarpa11_C/sagarpa11_C.html.

[25] Domínguez A. (1979). Methods of Phytochemical Research.

[26] Puerto N., Prieto G., Castro S. (2016). Proximal and physical-chemistry analysis of propolis from by boyacenses apiaries. Rev. De La Fac. De Cienc. Básicas.

[27] Sawyer R. (1988). Honey Identification.

[28] Erdtman G. (1969). An Introduction to the Study of Pollen Grains and Spores.

[29] Trigo M., Melgar M., García J., Recio M., Docampo S., Cabezudo B. (2007). Pollen in the atmosphere of Velez- Malaga. Velez Málaga, España: Concejalía del medio ambiente. Ayunt. Velez-Malaga.

[30] Singelton V., Orthofer R., Lamuela R. (1999). Analysis of Total phenol and other oxidation substrates and antioxidants by meanso Follin-Ciocalteau ragent. Method Enzymol..

[31] Ramamoorthy P., Bono A. (2007). Antioxidante activity, total phenolic and Flavonoid conted of Morinda citriflora fruit extracts from various extraction processes. J. Eng. Sci. Technol..

[32] Okusa P., Penge O., Devleeschouwer M., Dues P. (2007). Direct an indirect antimicrobial effects and antioxidant activity of Cordia gilleti De Wild (Borraginaceae). J. Ethnopharmacol..

[33] González M., Peñalosa C., Iztcala F. (2000). Biomolecules (Methods of Analysis).

[34] Jagota S., Dani H.A. (1982). New Colorometric Technique for the Estimation of Vitamin Cissing Phenol Reagan. Anal. Biochem..

[35] Chang R. (2007). Chemistry, México.

[36] Vanden D., Vlietnick K. (1991). Screening Methods for antibacterial angents from higher Plants. Methods Plant Biochem..

[37] Pritch G. (2019). Honey plants. Ediciones Omega.

[38] Cohen E., Rodríguez L.M. (2003). Guide to trees and shrubs in the metropolitan area of Mexico City. Gob. Del Dist. Federal. Secr. Del Medio Ambiente.

[39] (2018). NORMA Oficial Mexicana NOM-004-SAG/GAN-2018, Honey Production and Specifications. https://www.dof.gob.mx/nota_detalle.php?codigo=5592435&fecha=29/04/2020&print=true.

[40] Ciappini M., Gatti M., Di Vito M. (2013). Color as an indicator of flavonoids contení in honey. Cieñe. Tecnol..

[41] Vit P., Gutiérrez M.G., Titera D., Vendar M., Rodríguez A.J. (2008). Czech honey categorized according to their antioxidant activity, Acta Bioquím. Clin. Latinoam..

[42] Muñoz O., Ureta E., Peña R.C., Montenegro G. (2001). Propolis of matorral of Central Chile hives. Z. Naturforsch..

[43] Muñoz O., Peña R.C., Ureta E., Montenegro G., Caldwell C., Timmermann B.N. (2001). Phenolic compounds of propolis from central Chilean matorral. Z. Naturforsch..

[44] Dos Reis G.S., Valadão A.F., De Lima L.R.P., Moreira M.L. (2009). Preparación de un protector solar y evaluación de la acción fotoprotectora del propóleo verde del Vale do Aço, Minas Gerais, Brasil. Bol. Latinoam. Y Caribe Plantas Med. Y Aromat..

[45] Rosas A., Bautista M., Velázquez G. (2011). Atlas of the most important allergenic pollens in Mexico relevance in Mexico. Rev. Alerg. Mex..

[46] Montoya B.P., Baca A.E., Bonilla B.L. (2017). Melliferous flora and its offer of resources in five villages of the municipality of Piedamo, Cauca. La Rev. Biotecnol. En El Sect. Agropecu. Y Agroind..

[47] Cabrera-Pech J.V. (1966). Beekeeping and Beekeeping Flora in the Municipality Villa de Arriaga, S.L.P., Mexico. Bachelor’s Thesis.

[48] Ordetx G.S., Zosaya-Rubio J.A., Franco-Millán W. (1972). Study of the National Beekeeping Flora.

[49] Carmona M. (1980). Contribution to the Knowledge of the Melliferous Flora of the State of Morelos. Master’s Thesis.

[50] Bedascarrasbure E., Maldonado L., Alvarez A., Rodríguez E. (2004). Phenols and Flavonoids Content of Argentine Propolis. Acta Farm. Bonaer..

[51] Alejandre S., Ortiz M., Prieto A. (2021). Ecological Restoration in Times of COVID-19, Solution or Challenge?. Roca Rev. Científico Educ. Prov. Granma.

[52] Vargas C., Sánchez G., Jiménez P. (2013). The production of secondary metabolites in the brassicaceae family. Fac. Cienc. Basicas.

[53] Walker P., Crane E. (1987). Constituents of propolis. Apidologie.

[54] Salamanca G., Correa I.L., Principal J. (2007). Perfil de flavonoides e índices de oxidación de algunos propóleos colombianos. Zootec. Trop..

[55] White J.W., Kunter B., Abd Al-Manhel A.J., Rebersek P., Tascon N.N., Balan K., Omocea J., Keskin N. (1978). Honey. Advances in Food Research.

[56] White J.W., Crane E. (1979). Composition of honey. Honey: A Comprehensive Survey.

[57] White J.W., Gregor S.E. (1979). Composition and properties of honey. Beekeeping in the United States.

[58] BOE. (BOLETÍN OFICIAL DEL ESTADO) (2003). Real Decreto 1049/2003, de 1 de Agosto, 2003 por el que se Aprueba la Norma de Calidad Relativa a la miel. Madrid. España. https://www.boe.es/eli/es/rd/2003/08/01/1049.

[59] Crane E. (1990). The traditional hive products: Honey and beeswax. Bees and Beekeeping: Science, Practice and World Resources.

[60] Gonnet M., Opida I.N.R.A. (1982). Le Miel. Composition, Propriétés et Conservation.

[61] Molan P.C. (1992). The antibacterial activity of honey 1. The nature of the antibacterial activity. Bee World.

[62] Molan P.C. (1992). The antibacterial activity of honey 2. Variation in the potency of the antibacterial activity. Bee World.

[63] Tornuk F., Karaman S., Ozturk I., Toker O.S., Tastermur B., Sagdic O., Dogan M., Kayacier A. (2013). Quality characterization of artisanal and retail Turkish blossom honeys: Determination of physicochemical, microbiological, bioactive properties and aroma profile. Ind. Crops Prod..

[64] Estrada H., Gamboa M.M., Chaves C., Arias M.L. (2005). Evaluation of the antimicrobial activity of bee honey against *Staphylococcus aureus*, *Staphylococcus epidermidis*, *Pseudomonas aeruginosa*, *Escherichia coli*, Salmonella enteritidis, *Listeria monocytogenes* y *Aspergillus niger*. Arch. Latinoam. Nutr..

[65] Amaya M. (2009). Memory and learning in the floral choice of bees. Acta Biológica Colomb..

[66] Rodríguez M., Antonio J., Pérez P., Elizabeth M., Vit P. (2007). Antioxidant capacity of Venezuelan honeys of the Apis, Melipona and Tetragonisca genera, evaluated by three methods. Rev. Inst. Nac. Hig. Rafael Rangel.

[67] Ciappini M.C., Stoppani F.S., Martinet R., Alvarez M.B. (2013). Antioxidant activity and content of phenolic compounds and flavonoids in clover, eucalyptus, and alfalfa honeys. Rev. Cienc. Y Tecnol..

[68] White J.W., Subers M.H., Schepartz A.I. (1963). The identification of inhibine, the antibacterial factor in honey, as hydrogen peroxide and its origin in a honey glucose-oxidase system. Biochi. Biophys..

[69] Cruzado L., Gutiérrez D.P., Ruiz S.G. (2007). Chemical test and effect of antibiosis in vitro of the honey bee on gram-negative and gram-positive microorganisms. Rev. Med. Vallejiana.

[70] Bogdanov S. (1997). Nature and origin of the antibacterial substances in honey. Lebensm. Wiss Technol..

[71] Cabrera L., Céspedes E., Nava R., Ojeda G. (2006). Nonperoxide antibacterial activity of honeys from Zulia. Rev. Cient..

[72] Zamora L., Arias M. (2011). Microbial quality, and antimicrobial activity of stingless bee honey. Rev. Biomed..

[73] Eumkeb G., Siriwong S., Thumanu K. (2012). Synergistic activity of luteolin and amoxicilin combination against resistant *Escherichia coli* and mode of action. J. Photochem. Photobiol..

[74] Navarro J., Lezcano M.R., Mandri M.N., Gili M.A., Zamudio M.E. (2018). Anticariogenic action of propolis. RAAO.

[75] Garcia A., Ucar A., Ballester L. (2014). Elimination of candida albicans with ethanolic extract of commercial propolis of apis mellifera from the state of Merida, in partial denture bases. Removibles Rev. Odontol. Los Andes.

[76] Gil M., Joya M., Gonzales L., Figueroa Z., Perozo E. (2013). Fungistatic and Fungicidal Effect of Ethanolic Extract of Propolis on Candida Species.

